# Cardiovascular Toxicity in Cancer Therapy: Potential Mechanisms of Ferroptosis and Treatment Strategies

**DOI:** 10.3390/cells15171507

**Published:** 2026-08-22

**Authors:** Jiani Dai, Yufei Wang, Chunna Jin, Liuguang Song, Yao Xie, Liangliang Jia, Meixiang Xiang

**Affiliations:** 1 Department of Cardiology, The Second Affiliated Hospital, Zhejiang University School of Medicine, Hangzhou 310009, China; 22418420@zju.edu.cn (J.D.); 22318109@zju.edu.cn (Y.W.); 2316003@zju.edu.cn (C.J.); doctor_lgsong@zju.edu.cn (L.S.); xieyao@zju.edu.cn (Y.X.); 2 State Key Laboratory of Transvascular Implantation Devices, Hangzhou 310009, China; 3 Heart Regeneration and Repair Key Laboratory of Zhejiang Province, Hangzhou 310009, China; 4 Transvascular Implantation Devices Research Institute, Hangzhou 310053, China

**Keywords:** ferroptosis, cardio-oncology, cancer therapy, cardiovascular toxicity

## Abstract

**Highlights:**

**What are the main findings?**

- Ferroptosis exerts a dual role in cardio-oncology. Targeted ferroptosis eliminates drug-resistant tumors, while activation in cardiovascular system induces anticancer therapy-related cardiovascular toxicity.
- Chemotherapy, targeted therapy, immunotherapy, and radiotherapy induce cardiac ferroptosis through disrupted iron homeostasis and impaired multiple antioxidant defense pathways.

**What are the implications of the main findings?**

- This review establishes an integrated cardio-oncology framework linking tumor ferroptosis and cardiac ferroptosis, providing a mechanistic basis for balancing antitumor efficacy and cardiac safety.
- This review underscores an urgent need for the development of novel tissue-specific ferroptosis inhibitors and cardioprotective agents, as well as biomarker-based monitoring, to realize personalized management of anticancer therapy-related cardiovascular toxicity.

**Abstract:**

Advances in anticancer therapies have substantially improved cancer survival but have also highlighted the growing challenge of cancer therapy-related cardiac dysfunction. Ferroptosis, an iron-dependent form of regulated cell death characterized by iron dysregulation, lipid peroxidation, and impaired antioxidant defense, has emerged as a promising strategy for eliminating therapy-resistant tumors. However, the lack of tissue specificity in ferroptosis regulation raises concerns regarding its potential contribution to cardiovascular injury during anticancer treatment. This review summarizes the dual roles of ferroptosis in cancer biology and cardio-oncology. We first discuss the molecular mechanisms governing ferroptosis, including iron metabolism, lipid peroxidation, and antioxidant defense systems. We then highlight the context-dependent roles of ferroptosis in tumor progression, immune regulation, and metabolic adaptation. Furthermore, we systematically review how chemotherapy, targeted therapy, immunotherapy, and radiotherapy contribute to ferroptosis-associated cardiovascular toxicity. Finally, we discuss emerging approaches to minimize cardiac injury, including tissue-specific ferroptosis-targeting and cardioprotective strategies. Understanding tissue-specific ferroptosis regulation may facilitate the development of safer and more precise therapeutic approaches in cardio-oncology.

## 1. Introduction

Over the past three decades, the mortality rate of cancer has consistently declined. This improvement is partly due to the widespread secondary prevention, advances in diagnostic technologies, and new anticancer agents [1]. However, improved survival outcomes coupled with the cardiotoxic effects of these treatments have resulted in a growing population of cancer survivors with complications of cardiovascular diseases. Notably, evidence suggests that patients already exhibit a high prevalence of heart disease at initial cancer diagnosis, prior to treatment initiation [2]. Cardiovascular toxicity induced by anticancer therapies not only further impairs the quality of life in cancer patients, but also poses substantial challenges to clinicians. Thus, the discipline of cardio-oncology was established to understand, detect, and manage cardiovascular diseases induced by tumors and antitumor treatments [3].

Among the complex mechanisms of cancer therapy-related cardiac dysfunction (CTRCD), ferroptosis has recently been identified as a critical pathogenic process [4,5,6]. Ferroptosis is an iron-dependent form of regulated cell death characterized by excessive accumulation of lipid peroxides [7]. Its growing importance in oncology lies in targeting ferroptosis to eliminate cancer cells, especially those resistant to conventional therapies [8,9]. However, the mechanisms that induce ferroptosis in tumors—such as suppression of antioxidant defense systems, disruption of intracellular iron homeostasis, and promotion of lipid peroxidation—may similarly disrupt cardiac homeostasis [10,11,12,13]. With its high metabolic activity and abundant mitochondria, the heart is highly vulnerable to lipid peroxidation and iron-mediated oxidative injury. Consequently, ferroptosis is now considered a key driver in the pathogenesis of CTRCD [14].

This article reviews recent research advancements concerning the dual role of ferroptosis in tumor progression and anticancer treatment-induced cardiotoxicity. We aim to elucidate the molecular pathways by which ferroptosis contributes to anticancer therapy-induced cardiac injury, and to explore future research directions in this rapidly evolving field. A more profound understanding of ferroptosis may provide novel insights for cardioprotection and facilitate the development of more precise and safer anticancer therapies.

## 2. Molecular Mechanisms of Ferroptosis

**Figure 1:**
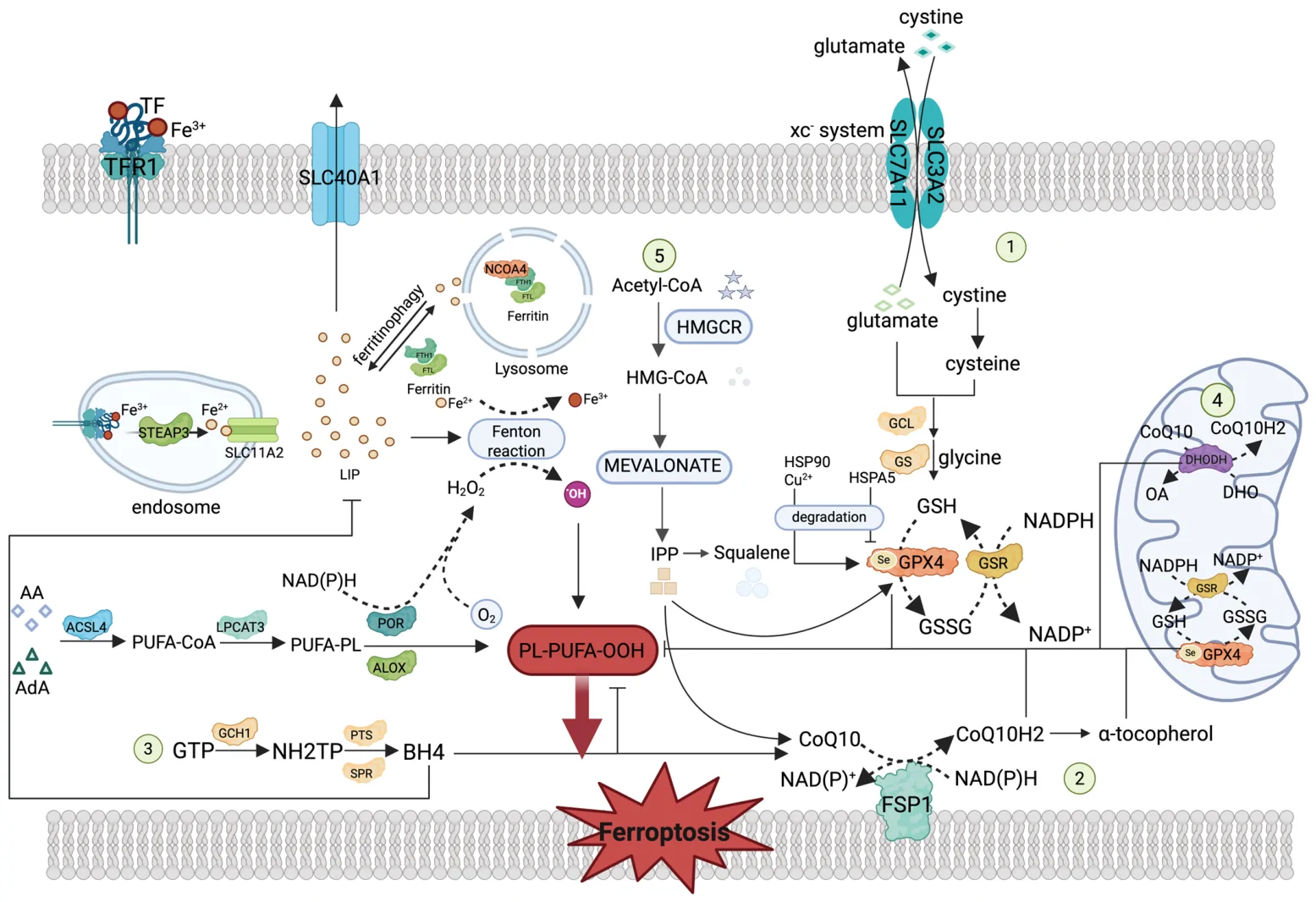
Core molecular machinery and signaling regulation of ferroptosis. The mechanisms of ferroptosis primarily involve three aspects: iron overload, impaired antioxidant defense, and dysregulated lipid metabolism. The uptake, export, and storage of intracellular iron are regulated by TFRC, FPN, and Ferritin, respectively; dysregulation of any of these can induce iron overload and promote ferroptosis via the Fenton reaction. AA and AdA are lipids most prone to oxidation. Under the catalysis of a series of enzymes (e.g., ACSL4, LPCAT3, ALOXs, POR), they are susceptible to lipid peroxidation, leading to ferroptosis. Key antioxidant systems—including system xc^−^–GSH–GPX4, FSP1–CoQ10–NAD(P)H, GCH1–BH4, mitochondrial DHODH and Mevalonate-CoQ10—protect against ferroptosis by reducing lipid peroxides to nontoxic alcohols. TFR1: Transferrin receptor; TF: Transferrin; STEAP3: Six-transmembrane epithelial antigen of prostate 3; SLC11A2: Solute carrier family 11 members 2; FTL: Ferritin light chain; FTH1: Ferritin heavy chain 1; NCOA4: Nuclear receptor co-activator 4; GCL: Glutamate–cysteine ligase; GS: Glutamine synthetase; GSH: glutathione; GSSG: Oxidized glutathione; GPX4: Glutathione peroxidase 4; HSP90: Heat shock protein 90; Cu^2+^: Copper(II) ion; HSPA5: Heat shock protein family A (Hsp70) member 5; GSR: Glutathione-disulfide reductase; AA: Arachidonic acid; AdA: Adrenic acid; ACSL4: Acyl-coenzyme A synthetase long-chain family member 4; LPCAT3: Lysophosphatidylcholine acyltransferase 3; ALOX: Arachidonate lipoxygenase; POR: Cytochrome P450 oxidoreductase; PUFA-CoA: Polyunsaturated fatty acyl-coenzyme A; PUFA-PL: Polyunsaturated fatty acid-containing phospholipid; GTP: Guanosine-5′-triphosphate; GCH1: GTP cyclohydrolase1; NH2TP: Dihydroneopterin triphosphate; PTS: 6-pyruvoyltetrahydropterin synthase; SPR: Sepiapterin reductase; BH4: Tetrahydrobiopterin; CoQ10: Ubiquinone; CoQ10H2: Ubiquinol; FSP1: Ferroptosis suppressor protein 1; SLC3A2: Solute carrier family 3 members 2. SLC7A11: Solute carrier family 7 member 11; SLC40A1: Solute carrier family 40 member 1; Se: Selenium; Fe^2+^: Ferrous iron; Fe^3+^: Ferric iron; OA: Orotate; DHO: Dihydroorotate; HMGCR: 3-hydroxy-3-methylglutaryl-coenzyme A reductase; HMG-CoA: 3-hydroxy-3-methylglutaryl-coenzyme A; IPP: Isopentenyl pyrophosphate; Acetyl CoA: Acetyl coenzyme A; PL-PUFA-OOH: Phospholipid-bound polyunsaturated fatty acid hydroperoxide; H_2_O_2_: Hydrogen peroxide; O_2_: Oxygen; •OH: Hydroxyl radical; NADP^+^: Nicotinamide adenine dinucleotide phosphate; NADPH: Nicotinamide adenine dinucleotide phosphate hydrogen. This figure was created in BioRender. WANG, Y. (2026) https://BioRender.com/ogudmao; accessed on 7 August 2026.

Ferroptosis is a distinct form of regulated cell death driven by iron-dependent phospholipid peroxidation, which sets it apart from apoptosis, necroptosis, and pyroptosis [15]. Unlike classical programmed cell death executed by dedicated protein machineries, ferroptosis arises from metabolic dysregulation—an imbalance between oxidative lipid damage and cellular antioxidant capacity [16]. Its execution is governed by three interconnected modules: iron metabolism, lipid peroxidation, and antioxidant defense [15]. Iron dysregulation expands the labile iron pool (LIP), fueling Fenton chemistry and reactive oxygen species (ROS) generation. Membrane phospholipids containing polyunsaturated fatty acids (PUFAs) undergo peroxidation, producing toxic lipid hydroperoxides that compromise membrane integrity [17]. To counteract these processes, cells have evolved multiple spatially segregated antioxidant defense systems—including the system xc^−^-GSH-GPX4 pathway, FSP1-CoQ10-NAD(P)H pathway, GCH1-BH4 pathway, mitochondrial DHODH pathway, and mevalonate-CoQ10 pathway—which collectively determine ferroptosis susceptibility. This section reviews the core molecular mechanisms of iron metabolism, lipid peroxidation, and antioxidant defense that underpin ferroptosis regulation (Figure 1).

### 2.1. Iron Metabolism

Iron overload is a fundamental characteristic of ferroptosis. The processes of cellular iron uptake, storage, metabolism, efflux, and turnover collectively regulate the cellular LIP, thereby influencing lipid peroxidation. Ferric iron (Fe^3+^), which is oxidized from ferrous iron (Fe^2+^) obtained through intestinal absorption or erythrocyte degradation, is recognized by transferrin and transported into cells via transferrin receptor (TFR1) [18]. It has been reported that hepatocyte-specific transferrin knockout mice are susceptible to ferroptosis-induced liver fibrosis [19]. Annexin A10, a member of the membrane-bound calcium-dependent phospholipid-binding protein family, confers ferroptosis resistance by facilitating autophagy-mediated TFR1 degradation, thereby promoting colorectal cancer progression [20]. Additionally, lactoferrin and its receptor act as positive regulators of ferroptosis by enhancing iron intake [21]. After absorption, Fe^3+^ is converted to Fe^2+^ by the metalloreductase six-transmembrane epithelial antigen of prostate 3 (STEAP3) within the endosome and subsequently transported into the cytosol by solute carrier family 11 member 2 (SLC11A2). Fe^2+^ serves multiple functions in metabolic and biochemical processes, primarily as a component of heme and iron–sulfur clusters, and is partially stored in ferritin and the LIP. Ferritin, the iron-storage protein, consists of ferritin light chain and ferritin heavy chain 1 (FTH1). It has been demonstrated that ferritin interacts with nuclear receptor coactivator 4 through the FTH1 subunit to form a complex. Autophagosomes containing the ferritin-nuclear receptor coactivator 4 complex are subsequently delivered to lysosomes for fusion, leading to ferritin degradation and subsequent iron release. This process, termed ferritinophagy, is activated during the induction of ferroptosis [22,23]. Furthermore, research indicates that the loss of FTH1 in cardiomyocytes can lead to cardiomyopathy via solute carrier family 7 member 11 (SLC7A11)-mediated ferroptosis [24]. Ferritin light chain downregulation-induced ferroptosis has been implicated in the pathogenesis of preeclampsia [25]. Ferroportin 1 (Fpn), also known as solute carrier family 40 member 1, is the only identified mammalian nonheme cellular iron exporter to date [26]. Fpn transports iron from iron-storing cells into the blood to optimize systemic iron homeostasis. Knockdown of Fpn leads to an abnormal accumulation of Fe^2+^ within the intracellular LIP, which intensifies the Fenton reaction and results in excessive ROS production, thereby promoting ferroptosis [27,28]. Dysregulation of Fpn has been implicated in multiple diseases, including cardiac dysfunction, acute respiratory distress syndrome, and chemoresistance, via the disruption of iron homeostasis [28,29,30].

### 2.2. Lipid Peroxidation

Lipid peroxidation is a free radical-driven reaction that predominantly affects PUFAs. This reaction can occur on both free PUFAs and PUFA-containing membrane phospholipids. Arachidonic acid (AA) and adrenic acid (AdA) are significant PUFAs, and their peroxidation at the bis-allylic position plays a critical role in the promotion of ferroptosis [17].

It has been observed that peroxidation can be triggered by both enzymatic processes and nonenzymatic autoxidation. In eukaryotic cells, acyl-coenzyme A synthetase long-chain family member 4 (ACSL4) activates PUFAs to generate PUFA-CoA, which is then integrated into the phospholipids of the plasma membrane through the action of lysophosphatidylcholine acyltransferase 3 (LPCAT3). This incorporation results in the formation of PUFA-containing membrane phospholipids [31,32]. Tumor cell ferroptosis induced by the concurrent presence of AA and interferon-γ (IFN-γ) can be abolished by the absence of ACSL4, indicating that ACSL4 is a pivotal enzyme in ferroptosis mediated by the combination of AA and IFN-γ [33]. The mammalian arachidonate lipoxygenase (ALOX) family, comprising six members (e.g., ALOXE3, ALOX5, ALOX12, ALOX12B, ALOX15, ALOX15B), plays a tissue- or cell-dependent role in oxidizing phosphatidylethanolamines containing PUFA into detrimental lipid hydroperoxide products, such as PE-AA-OOH, thereby inducing ferroptosis. It has been reported that at least three lipoxygenase enzymes (e.g., ALOX5, ALOX12, ALOX15) are involved in the catalysis of phospholipid hydroperoxide generation during the ferroptosis process [34,35,36]. ALOX15-targeting small activating RNA-loaded mesoporous polydopamine coated with angiopep-2-modified macrophage membranes was designed to treat orthotopic glioblastoma-bearing mice, exhibiting a marked inhibitory effect on glioblastoma progression and a promoting effect on glioblastoma radiosensitivity [37]. In addition to ACSL4 and the ALOX family, cytochrome P450 reductase has been reported as an oxidoreductase that transfers electrons from NADH/NADPH to oxygen, generating hydrogen peroxide and consequently triggering lipid peroxidation.

**Figure 2:**
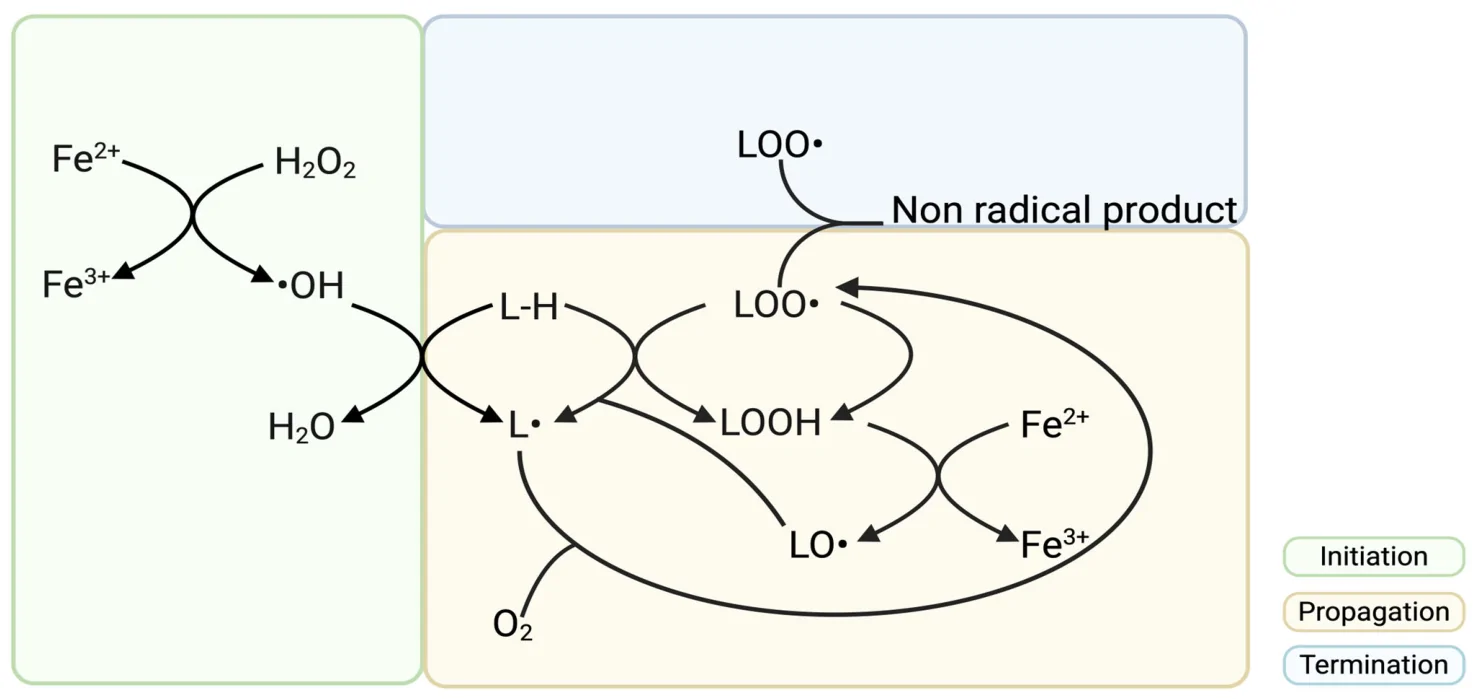
Lipid peroxidation mediated by autoxidation includes three steps: initiation, propagation, and termination. Fe^2+^: Ferrous iron; Fe^3+^: Ferric iron; H_2_O_2_: Hydrogen peroxide; •OH: Hydroxyl radical; L-H: Polyunsaturated fatty acids; L•: Lipid radical; LOO•: Lipid peroxyl radical; LOOH: Lipid hydroperoxide; LO•: Lipid alkoxyl radical; O_2_: Oxygen; H_2_O: Dihydrogen monoxide. This figure was created in BioRender. WANG, Y. (2026) https://BioRender.com/28ah5ft; accessed on 7 August 2026.

Lipid peroxidation mediated by autoxidation mainly encompasses three principal steps: initiation, propagation, and termination [38]. During this process, lipid peroxides engage with Fe^2+^ to generate an alkoxyl radical, which subsequently reacts with oxygen to yield a peroxyl radical capable of abstracting hydrogen from adjacent acyl chains within the lipid membrane milieu, thus propagating the lipid peroxidation process [39,40]. These propagation reactions compete with the termination phase, wherein two peroxyl radicals interact to generate nonradical products such as an alcohol, a carbonyl compound, and oxygen. Ron Shah et al. elucidated that lipoxygenases are instrumental in the initiation of ferroptosis by augmenting the cellular pool of lipid hydroperoxides that facilitate lipid autoxidation [41] (Figure 2).

### 2.3. Antioxidant Defense

#### 2.3.1. System xc^−^-GSH-GPX4 Pathway

In the context of antioxidant defense against ferroptosis, the system xc^−^-GSH-GPX4 pathway plays a pivotal role. This amino acid antiporter system, which consists of the light-chain subunit SLC7A11 and the heavy-chain subunit solute carrier family 3 member 2 (SLC3A2), functions by exchanging extracellular cystine for intracellular glutamate, thereby supporting the synthesis of glutathione (GSH). GSH serves as a potent antioxidant and is a cofactor for GSH peroxidase 4 (GPX4). As a phospholipid hydroperoxidase, GPX4 inhibits ferroptosis by reducing phospholipid hydroperoxides to the corresponding phospholipid alcohols, while GSH is oxidized to GSH disulfide. In addition to GSH, the expression and activity of GPX4 can also be regulated by selenium. During GPX4 synthesis, the nascent polypeptide chain incorporates selenium as selenocysteine, where selenium replaces the sulfur atom of cysteine. This incorporation occurs when a UGA stop codon is “recoded” by a selenocysteine-tRNA and a selenocysteine insertion sequence within the GPX4 mRNA. Selenium can enhance the anti-ferroptosis activity of GPX4 through a selenocysteine residue at position 46 [42]. Although the anti-ferroptotic function of GPX4 has been extensively explored, the specific mechanisms underlying GPX4 degradation that promote ferroptosis remain poorly understood. Heat shock proteins have been identified as key regulators of GPX4 protein degradation. For instance, heat shock protein 90 (HSP90) functions as a molecular chaperone to facilitate the degradation of GPX4 via chaperone-mediated autophagy, whereas heat shock protein family A (Hsp70) member 5 (HSPA5) acts to protect against the protein degradation of GPX4 [43,44]. Notably, copper can directly bind to GPX4 and induce its subsequent autophagic degradation via Tax1 binding protein 1, further amplifying ferroptotic cell death in pancreatic cancer tumors [45]. Recent studies have identified several small molecules and natural products, including DMOCPTL (a synthetic derivative of parthenolide) and bufadienolides, that can induce tumor cell ferroptosis by facilitating the ubiquitination and degradation of GPX4 [46,47] (Figure 1).

#### 2.3.2. FSP1-CoQ10-NAD(P)H Pathway

Ferroptosis suppressor protein 1 (FSP1), initially described as apoptosis-inducing factor mitochondrial 2, was renamed after being identified as a GSH-independent inhibitor of ferroptosis that functions in parallel to the GSH-dependent GPX4 pathway [48]. The myristoylation motif at the N-terminus of FSP1 facilitates its localization to the plasma membrane, which is required for its protective role against ferroptosis. Subsequently, plasma membrane-localized FSP1 reduces ubiquinone (also called CoQ10) to ubiquinol by consuming NADH/NADPH [49]. Ubiquinol acts as a lipophilic antioxidant that captures free radicals to prevent lipid peroxidation and can also indirectly regenerate another antioxidant, α-tocopherol, which captures free radicals to inhibit ferroptosis. Inhibiting FSP1 is regarded as a promising strategy to counteract ferroptosis resistance in multiple cancer types. However, due to its unfavorable structure and limited potential for medicinal chemistry development, the first identified specific inhibitor of FSP1, FSP1 inhibitor, is not suitable for further development as an anticancer drug [50]. Recently, a new class of compounds known as 3-phenylquinazolinones, which are activators of FSP1 condensation, have been identified as next-generation FSP1 inhibitors. These compounds trigger ferroptosis through the dissociation of FSP1 from the membrane and the formation of FSP1 condensates via phase separation [51]. Moreover, the ability of FSP1 condensation to inhibit tumor growth and reduce tumor weight has been confirmed in a tumor-bearing mouse model (Figure 1).

#### 2.3.3. GCH1-BH4 Pathway

A genome-wide activation screen analysis has revealed that GTP cyclohydrolase-1 (*GCH1*) is a novel gene that robustly regulates ferroptosis [52]. The increased cellular level of GCH1 is accompanied by a significant elevation of its downstream metabolites, primarily tetrahydrobiopterin (BH4) and dihydrobiopterin. GCH1 catalyzes the first and rate-limiting step in converting guanosine-5′-triphosphate (GTP) to 7,8-dihydroneopterin triphosphate (NH2TP). Two other enzymes, namely, 6-pyruvoyltetrahydropterin synthase (PTS) and sepiapterin reductase (SPR), subsequently convert 7,8-dihydroneopterin triphosphate (NH2TP) to BH4. BH4 regulates ferroptosis sensitivity by trapping lipid-derived peroxyl radicals and inhibiting lipid peroxidation, independent of its role as a redox-active cofactor for several biosynthetic enzymes, including nitric oxide synthase, alkylglycerol monooxygenase, and aromatic amino acid hydroxylases [53]. The antioxidant activity of BH4 may be particularly important for T-cell function, as its availability has been reported to alter iron metabolism and mitochondrial function in T cells [54]. In addition, cells overexpressing GCH1 also showed a significant increase in CoQ10 levels, whose biosynthesis is substantially supported by BH4 [52]. In conclusion, the BH4 biosynthesis pathway serves as a critical regulator of tumor cell sensitivity to ferroptosis. Recently, a novel circular RNA, circLRFN5, has been identified as being downregulated in glioblastoma, suggesting that it promotes gliomagenesis through the paired related homeobox 2 (PRRX2)/GCH1/BH4 signaling axis [55] (Figure 1).

#### 2.3.4. Mitochondrial DHODH Pathway

Dihydroorotate dehydrogenase (DHODH) is an inner mitochondrial membrane flavoprotein involved in de novo pyrimidine biosynthesis, catalyzing the oxidation of dihydroorotate to orotate while transferring electrons to CoQ10 in the mitochondrial respiratory chain [56]. Beyond its canonical metabolic function, DHODH has recently emerged as a mitochondrial ferroptosis suppressor that functions in parallel with the cytosolic GPX4 and plasma membrane-associated FSP1 systems [56,57]. Mechanistically, DHODH maintains mitochondrial CoQ10/ubiquinol (CoQH2) redox cycling, thereby preserving mitochondrial antioxidant capacity and limiting mitochondrial lipid peroxidation. CoQH2, also called reduced CoQ10, functions as a lipid-soluble radical-trapping antioxidant that scavenges lipid peroxyl radicals and prevents the propagation of lipid peroxidation [56]. Notably, the DHODH-CoQH2 pathway confers exclusive mitochondria-specific ferroptosis resistance, which is functionally distinct from the FSP1-CoQ10-NAD(P)H pathway that primarily safeguards cytoplasmic membrane integrity (Figure 1).

#### 2.3.5. Mevalonate-CoQ10 Pathway

The mevalonate (MVA) pathway, a central metabolic cascade driving cholesterol and isoprenoid biosynthesis, has been recognized as an upstream regulator of ferroptosis sensitivity. Through the generation of isopentenyl pyrophosphate (IPP), the MVA pathway provides essential precursors for CoQ10 biosynthesis and maintains cellular antioxidant capacity [16,58]. CoQ10 functions as both a mitochondrial electron carrier and a lipid-soluble radical-trapping antioxidant that suppresses lipid peroxidation and ferroptosis [16]. Mechanistically, the MVA pathway regulates ferroptosis resistance through two interconnected processes: promoting CoQ10 synthesis and supporting selenocysteine-tRNA maturation required for GPX4 translation. Clinically, statins inhibit 3-hydroxy-3-methylglutaryl coenzyme A (HMG-CoA) reductase, the rate-limiting enzyme of the MVA pathway, thereby reducing downstream isoprenoid metabolites and CoQ10 availability [58]. Accumulating evidence indicates that statin-mediated MVA-pathway suppression impairs both CoQ10-dependent and GPX4-dependent ferroptosis defense, rendering cells more vulnerable to ferroptosis [58,59] (Figure 1).

## 3. Role of Ferroptosis in Cancer

Ferroptosis acts as a double-edged sword in cancer biology, exerting context-dependent tumor-suppressive and tumor-promoting effects. Inducing ferroptosis in tumor cells provides a compelling therapeutic strategy, especially for eliminating tumors that are resistant to conventional apoptosis-based treatments. Conversely, ferroptosis occurring within the tumor microenvironment (TME) remodels immune cell infiltration and metabolic homeostasis, thereby favoring tumor immune evasion and malignant progression [60]. This section reviews the multifaceted roles of ferroptosis in tumor progression from three perspectives: tumor protein p53 (p53)-mediated regulation, immune regulation within the TME, and metabolic rewiring that determines ferroptosis sensitivity.

### 3.1. p53 Mediated Ferroptosis

Cellular death serves as a physiological regulator of cell proliferation, with both processes exerting profound influences on growth and development throughout an organism’s life. Reports indicate that an increased rate of cell proliferation and abnormalities within the cell cycle can result from the inactivation of tumor suppressor genes such as *p53* [61,62]. p53 is recognized as a tumor suppressor protein that possesses multiple and robust functions, including the arrest of the cell cycle, the initiation of cell death, and the repair of DNA damage. The regulation of ferroptosis by p53 is multifaceted, context-dependent, and varies by tissue type. p53 can transcriptionally inhibit the expression of SLC7A11, leading to GSH depletion, thereby enhancing the sensitivity of cancer cells to ferroptosis in colorectal cancer, chronic lymphocytic leukemia, and esophageal cancer [63,64]. In addition to its effects on SLC7A11, p53 increases levels of ALOX15, which oxygenates PUFAs to promote ferroptosis via targeting spermidine/spermine N1-acetyltransferase and enhance its expression [65]. Moreover, p53 can also induce the expression of lncRNA plasmacytoma variant translocation 1, which functions as a molecular sponge for microRNA miR-214, leading to its sequestration and functional repression, thereby promoting ferroptosis by restraining the miR-214-mediated downregulation of TFR1 [66].

**Figure 3:**
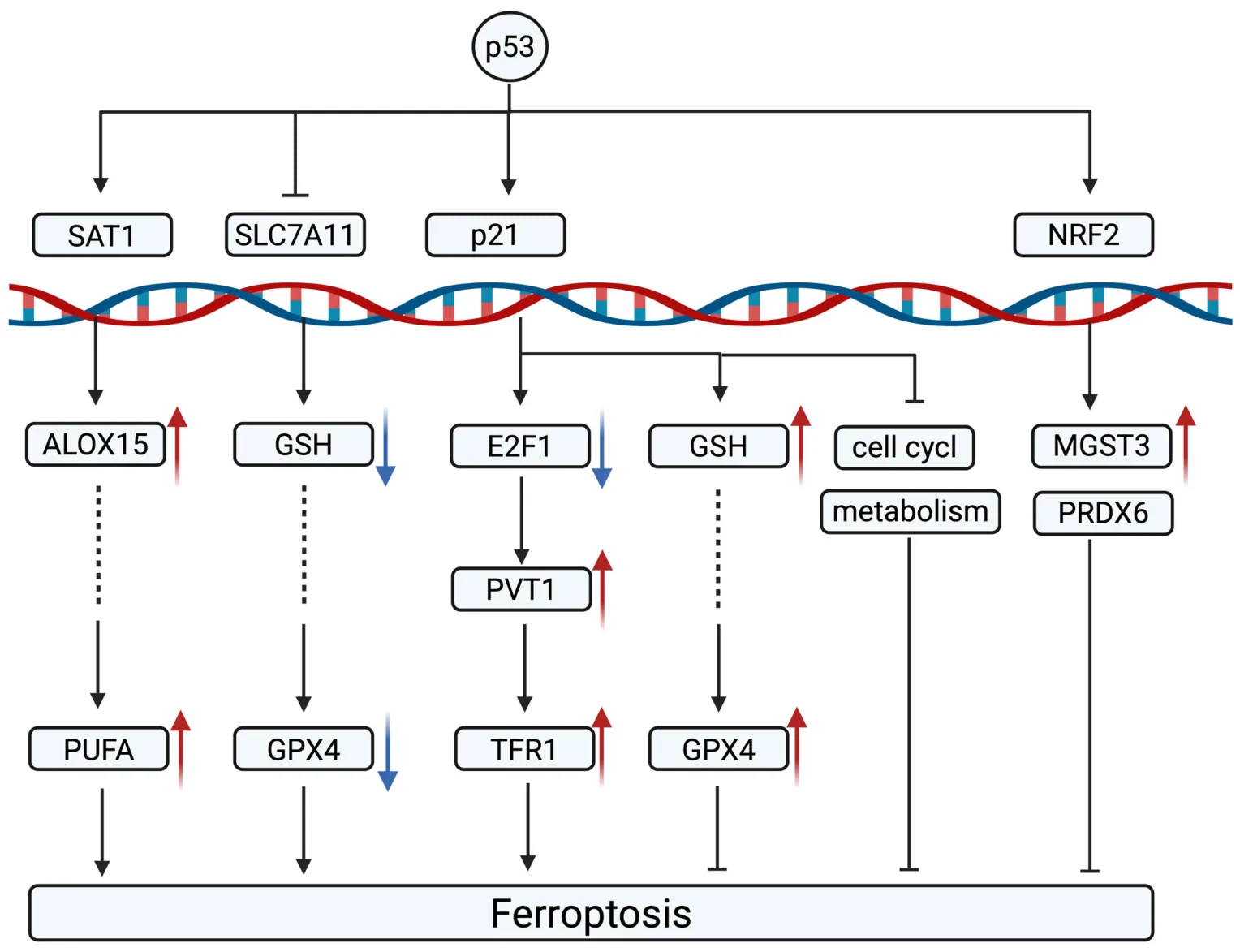
Role of p53 in ferroptosis. p53 play a dual role in ferroptosis. On one hand, p53 enhance ferroptosis by SAT1/ALOX15 pathway, SLC7A11/GSH/GPX4 pathway, and p21/E2F1/PVT1/TFR1 pathway; On the other hand, p53 inhibit ferroptosis by p21/GSH/GPX4 pathway and NRF2/MGST3, PRDX6 pathway. SAT1: Spermidine/Spermine N^1^-acetyltransferase 1; ALOX15: Arachidonate 15-lipoxygenase; SLC7A11, Solute carrier family 7 member 11; GPX4: Glutathione peroxidase 4; GSH: Glutathione; E2F1: E2F transcription factor 1; PVT1 lncRNA: Plasmacytoma variant translocation 1 long noncoding RNA; TFRC: Transferrin receptors; NRF2: Nuclear factor erythroid 2-related factor 2; MGST3: Microsomal glutathione S-transferase 3; PRDX6: Peroxiredoxin 6; p53: Tumor protein p53; p21: Cyclin-dependent kinase inhibitor 1A; PUFA: Polyunsaturated fatty acid; TFR1: Transferrin receptor. This figure was created in BioRender. WANG, Y. (2026) https://BioRender.com/g9xdwxe; accessed on 7 August 2026.

Despite p53’s well-established role in promoting ferroptosis, numerous studies have highlighted its context-dependent role as a key suppressor of ferroptosis. The core mechanism by which p53 inhibits ferroptosis involves the transcriptional activation of its critical downstream target, cyclin-dependent kinase inhibitor 1A (also called p21) [67]. The induction of p21 triggers robust G1 cell cycle arrest, leading to a comprehensive decrease in metabolic activity, characterized by reduced lipid synthesis and diminished ROS generation, thereby mitigating the fundamental drivers of ferroptosis. Additionally, p21 upregulates GSH biosynthesis, providing adequate cofactors for GPX4 to reinforce lipid peroxide reduction and strengthen the cellular antioxidant defense mechanisms [68]. More importantly, in a clinical context, p53 gain-of-function mutants exhibit a remarkable capacity to suppress ferroptosis. These mutants enhance the transcriptional activity of nuclear factor erythroid 2-related factor 2 (NRF2), driving the expression of a range of antioxidant genes, such as microsomal GSH S-transferase 3 (*MGST3*) and peroxiredoxin 6 (*PRDX6*), which encode two GSH-dependent peroxidases, thus suppressing ferroptosis [69] (Figure 3).

### 3.2. Immune Regulation of Ferroptosis

Tumor-associated macrophages (TAMs) are the predominant immune cell type within the TME, encompassing both M1 macrophages, which exhibit antitumor properties, and M2 macrophages, which are associated with tumor promotion. It is reported that 27-hydroxycholesterol, a primary metabolite of cholesterol, inhibits ferroptosis in hepatocellular carcinoma (HCC) cells. It is extruded from cells to facilitate the metabolic reprogramming of TAMs by activating the peroxisome proliferator-activated receptor-γ (PPAR-γ)/carnitine palmitoyltransferase 1A (CPT1A) signaling pathway, thereby encouraging M2 polarization [70]. Additionally, KRASG12D (oncogenic *Kras^G12D^* mutation protein) acts as a damage-associated molecular pattern that is released from exosomes of ferroptotic pancreatic ductal adenocarcinoma (PDAC) cells. When macrophages uptake this damage-associated molecular pattern via the advanced glycosylation end-product-specific receptor pathway, it triggers M2 polarization in macrophages by activating signal transducer and activator of transcription 3 (STAT3)-dependent fatty acid oxidation [71]. Conversely, itaconate, primarily derived from TAMs, is taken up by tumor cells via solute carrier family 13 member 3 (SLC13A3). Itaconate directly alkylates specific cysteine residues on the Kelch-like ECH-associated protein 1 (KEAP1), thereby protecting NRF2 from ubiquitination-mediated degradation [72]. This stabilization facilitates NRF2-mediated transcriptional activation of antioxidant genes (e.g., *SLC7A11*, *GPX4*), consequently conferring ferroptosis resistance to tumor cells.

**Figure 4:**
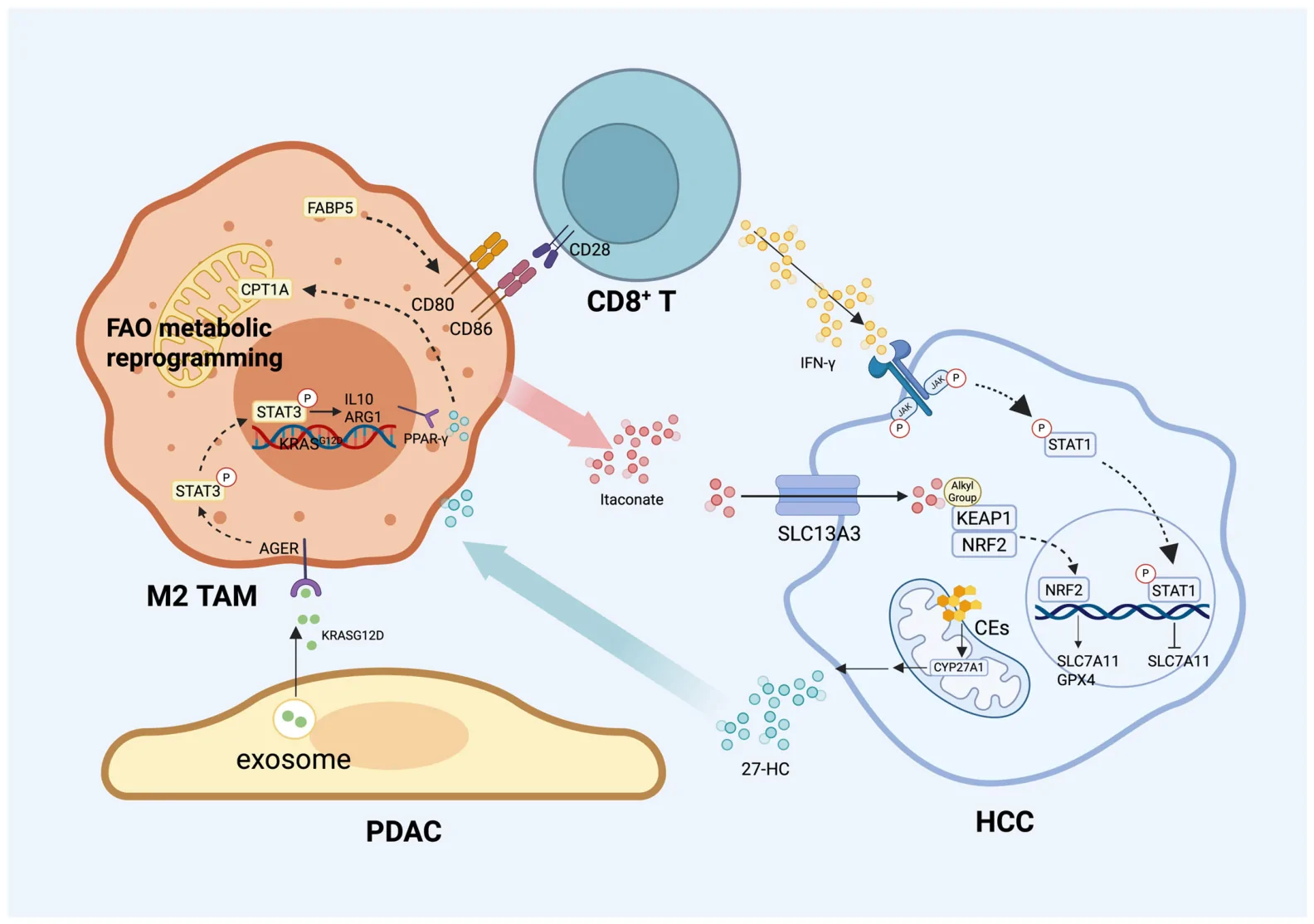
Role of ferroptosis in tumor immunity. HCC-derived 27-HC suppresses ferroptosis in cancer cells while promoting the polarization of pro-tumorigenic M2 macrophages. Ferroptotic PDAC cells release KRASG12D, which also drives TAMs toward M2 polarization. Conversely, itaconate secreted by TAMs inhibits tumor cell ferroptosis via the KEAP1/NRF2 pathway. Additionally, TAM-mediated activation of CD8^+^ T cells induces IFN-γ secretion, leading to ferroptosis in tumor cells. FAO: Fatty acid oxidation; FABP5: Fatty acid-binding protein 5; 27-HC: 27-Hydroxycholesterol; PPAR-γ: Peroxisome proliferator-activated receptor-γ; CPT1A: Carnitine palmitoyltransferase 1 A; AGER: Advanced glycation end-product receptor; STAT3: Signal transducer and activator of transcription 3; IL10: Interleukin 10; ARG1: Arginase 1; SLC13A3: Solute carrier family 13 member 3; IFN-γ: Interferon-γ; JAK: Janus kinase; STAT1: Signal transducer and activator of transcription 1; KEAP1: Kelch-like ECH-associated protein 1; NRF2: Nuclear factor erythroid 2-related factor 2; CEs: Cholesterol esters; CYP27A1: Cytochrome p450 family 27 subfamily A member 1; TAM: Tumor-associated macrophage; KRASG12D: *Kras^G12D^* mutation protein; PDAC: Pancreatic ductal adenocarcinoma; HCC: Hepatocellular Carcinoma; SLC7A11: Solute carrier family 7 member 11; GPX4: Glutathione peroxidase 4; P: Phosphorylation. This figure was created in BioRender. WANG, Y. (2026) https://BioRender.com/m3ke2ga; accessed on 7 August 2026.

In addition to the communication between TAMs and ferroptotic cancer cells, interplay among diverse tumor niche components adds an additional layer of complexity to the TME. Sun et al. have demonstrated that fatty acid-binding protein 5 (FABP5) is a critical regulator of macrophage-induced T cell activation by controlling the surface expression of co-stimulatory ligands CD80 and CD86 [73]. These ligands trigger CD28 receptor activation in CD8^+^ T cells, thereby enhancing the proliferation and cytotoxicity of tumor-infiltrating T cells [73]. It has been reported that CD8^+^ T cells secrete IFN-γ, which facilitates ferroptosis in tumor cells by inhibiting SLC7A11 through a signal transducer and activator of transcription 1 (STAT1)-mediated mechanism and establishing crosstalk between the IFN-γ and ACSL4 signaling pathways [74,75] (Figure 4).

### 3.3. Metabolic Regulation of Ferroptosis

Given the inherently metabolic nature of ferroptosis, it is unsurprising that this phenomenon is intricately regulated by a variety of critical metabolic elements. Reprogrammed metabolism and metabolic signaling in cancer cells favor escape from cell death by transforming the TME, thereby suppressing the anti-tumor immune response. Recent findings have illustrated that cancer cells, in conjunction with myeloid cells, consume the majority of available glucose in the TME. Due to glucose scarcity in the TME, cancer cells deplete substantial adenosine triphosphate reserves, which catalyzes activation of the adenosine monophosphate-activated protein kinase pathway. This in turn suppresses the synthesis of PUFAs, consequently imparting resistance to ferroptosis [76]. Additionally, cancer cells intensively utilize anaerobic glycolysis, resulting in extracellular acidification due to the production and release of substantial amounts of lactic acid [77]. This acidic microenvironment not only promotes tumor metastasis but also inhibits ferroptosis in tumor cells through the hydroxycarboxylic acid receptor 1/monocarboxylate transporter 1/sterol regulatory element-binding protein 1/stearoyl-CoA desaturase 1 (HCAR1/MCT1/SREBP1/SCD1) pathway, facilitating metastatic potential and tumor development [78].

## 4. Cardiac Ferroptosis Caused by Anticancer Treatment

**Figure 5:**
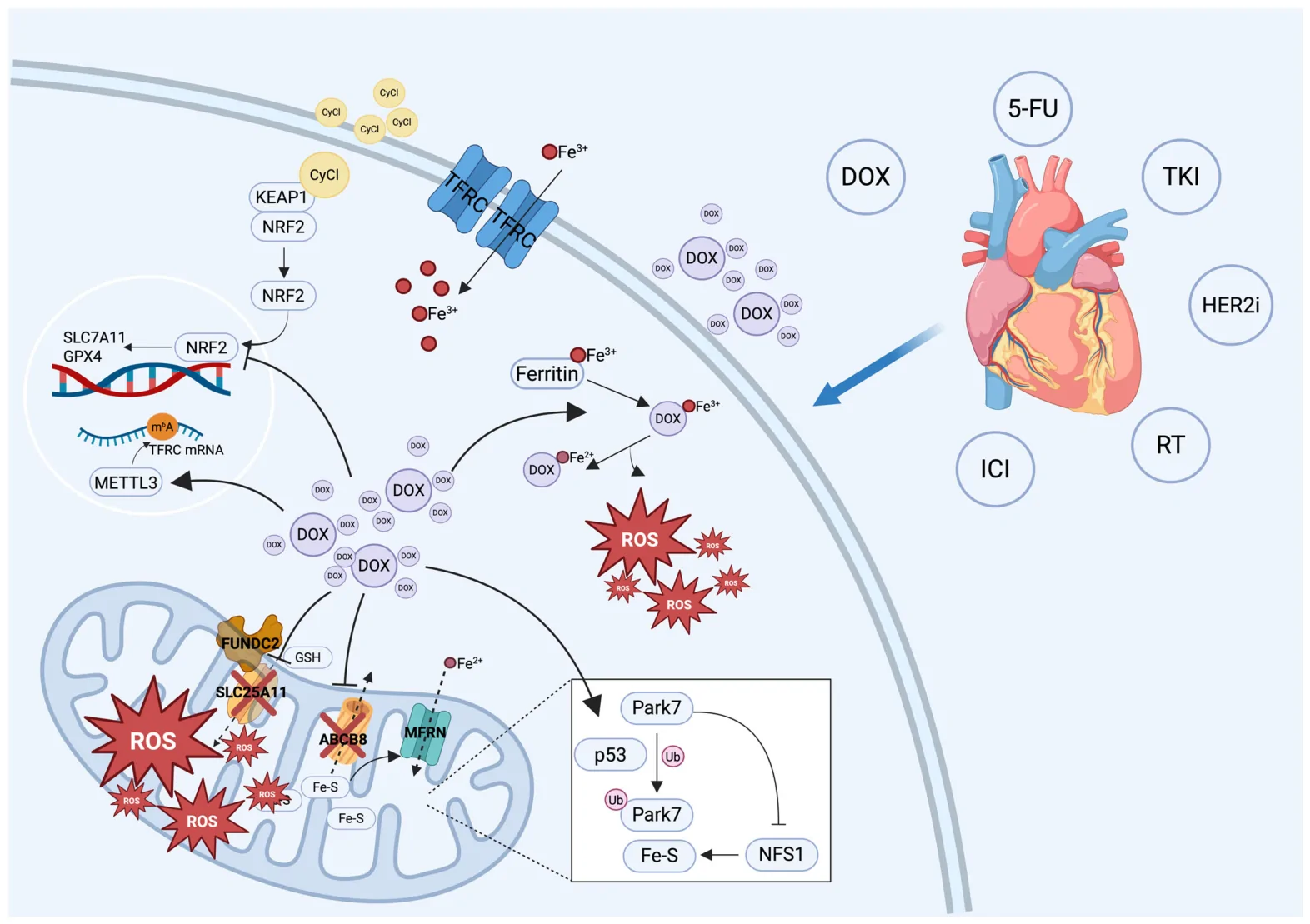
Role of ferroptosis in DOX-induced cardiomyopathy. Mechanisms of DOX-induced cardiomyocyte ferroptosis include antioxidant system disruption and iron overload. DOX impairs mitochondrial GSH uptake by destabilizing SLC25A11 via FUNDC2 interaction and suppresses antioxidant gene expression by inhibiting the NRF2/KEAP1 pathway. Concurrently, it downregulates ABCB8 to inhibit iron export, promotes p53-mediated Park7 degradation leading to compensatory iron–sulfur cluster overproduction, and enhances TFRC translation via METTL3-dependent m^6^A methylation. In addition, DOX directly extracts iron from ferritin. Abbreviations: METTL3: Methyltransferase-like 3; FUNDC2: FUN14 domain-containing 2; ABCB8: ATP-binding cassette subfamily B member 8; Fe-S: Iron–sulfur cluster; MFRN: Mitoferrin; NFS1: Nitrogen fixation 1; Park7: Parkinson’s disease protein 7; CyCl: Cyanidin chloride; KEAP1: Kelch-like ECH-associated protein 1; NRF2: Nuclear factor erythroid 2-related factor 2; TFRC: Transferrin receptors; DOX: Doxorubicin; 5-FU: 5-Fluorouracil; TKI: Tyrosine kinase inhibitor; HER2i: HER2 inhibitors; RT: Radiation therapy; ICI: Immune checkpoint inhibitor; p53: Tumor protein p53; Fe^3+^: Ferric iron; Fe^2+^: Ferrous iron; ROS: Reactive oxygen species; SLC7A11: Solute carrier family 7 member 11; GPX4: Glutathione peroxidase 4; SLC25A11: Solute carrier family 25 member 11; Ub: Ubiquitination. This figure was created in BioRender. WANG, Y. (2026) https://BioRender.com/nllaeyk; accessed on 7 August 2026.

Given the dual regulatory role of ferroptosis in tumor progression and cancer treatment, ferroptosis-inducing anticancer strategies act as a double-edged sword [60]. While triggering ferroptosis in malignant cells provides a viable approach to eradicating drug-resistant tumors, such therapeutic interventions often cause off-tumor damage to normal tissues. The heart is particularly susceptible to ferroptotic injury due to its abundant mitochondrial content, high iron dependence, and elevated oxygen consumption [79,80,81]. The resultant cardiovascular damage, collectively defined as cancer therapy-related cardiovascular toxicity, manifests as multiple adverse conditions, including heart failure, hypertension, myocarditis, and atherosclerosis [82]. This section systematically summarizes the ferroptosis-driven mechanisms underlying cardiovascular toxicity induced by mainstream anticancer modalities, including chemotherapy, targeted therapy, immunotherapy, and radiotherapy, examples are provided in Table 1 (Figure 5). Of particular note, we highlight the roles of the crosstalk between endoplasmic reticulum (ER) stress and ferroptosis, as well as metabolic regulation-mediated ferroptosis, in mediating anticancer therapy-related cardiotoxicity.

### 4.1. Anthracycline-Induced Cardiac Ferroptosis

Anthracyclines, such as doxorubicin (DOX) and daunorubicin, are widely utilized as broad-spectrum anticancer agents for the treatment of diverse malignancies. However, their clinical application is often hindered by severe cardiotoxicity. Anthracyclines-induced cardiotoxicity remains a significant contributor to heart disease-related mortality among cancer survivors and has attracted increasing attention from clinicians. Here, this review employs DOX as a representative example to illustrate these concerns.

#### 4.1.1. Dysregulation of Antioxidant Defense

Various mechanisms contribute to DOX-induced cardiomyopathy (DIC), among which ferroptosis has attracted considerable attention. It is reported that FUN14 domain-containing 2, a highly conserved and ubiquitously expressed mitochondrial outer membrane protein, interacts with solute carrier family 25 members 11 (SLC25A11) and destabilizes it under DOX treatment, thereby destabilizing mitochondrial GPX4 and triggering ferroptosis in cardiomyocytes [83]. Selenomethione (SeMet), a GPX4 activator, suppresses DOX-induced cardiomyocyte ferroptosis in a GPX4-dependent manner. Clinically well-tolerated and without impairing chemotherapy efficacy, it represents a promising therapeutic for preventing DIC [84]. The NRF2/KEAP1 pathway is a crucial signaling axis in counteracting DOX-induced ferroptosis in cardiomyocytes. Through high-content phenotypic screening, Liu et al. have identified cyanidin chloride as a potent inhibitor of DIC, which primarily targets the NRF2/KEAP1 pathway. Molecular docking and thermal shift assay further implicated a direct binding between cyanidin chloride and KEAP1, which promotes nuclear accumulation of NRF2 by dissociating NRF2 from KEAP1. The cardiac protective role of cyanidin chloride has been validated in a mouse model of DIC [85].

#### 4.1.2. Excessive Iron Accumulation

DOX shows a particular predilection for mitochondria in cardiomyocytes, leading to iron accumulation in the mitochondria by downregulating ABC protein-B8 and inhibiting the activity of iron regulatory protein 1 and iron regulatory protein 2, which are located on mitochondria and involved in iron regulation [86,87,88]. Previous studies also revealed that DOX can directly extract Fe^3+^ from ferritin, resulting in the formation of a DOX-Fe^3+^ complex, which is then reduced to DOX-Fe^2+^. This iron cycling between the Fe^2+^ and Fe^3+^ contributes to additional ROS production [89]. Moreover, upon DOX challenge, parkinsonism-associated deglycase (DJ-1) is ubiquitinated and degraded in a p53-dependent manner. This degradation results in a compensatory induction of mitochondrial iron–sulfur cluster formation via mitoferrin, leading to mitochondrial iron overload and myocardial ferroptosis [90]. Dexrazoxane (DXZ) has been approved by the Food and Drug Administration (FDA) for the treatment of DIC. It can effectively reverse the cardiotoxic effects of DOX by reducing mitochondrial iron levels [91,92]. In contrast, deferoxamine, a membrane-impermeable iron chelator that binds extracellular free Fe^3+^, is not effective in preventing DIC. This is partially because DOX-Fe^2+^, rather than Fe^3+^, accumulates in mitochondria of cultured cardiomyocytes and plays a central role in DOX-related lipid peroxidation [92]. Wu et al. have elucidated that the methyltransferase-like 3 (METTL3)-mediated m^6^A modification of TFR1 mRNA is elevated in cardiomyocytes to induce DIC by promoting cardiac iron uptake and ferroptosis. A highly selective methyltransferase-like 3 inhibitor, STM 2457, has displayed therapeutic benefits in malignancies (e.g., myeloid leukemia, small-cell lung cancer, HCC) and is found to significantly attenuate DOX-induced cardiac dysfunction in a chronic mouse model [93].

### 4.2. 5-FU-Induced Ferroptosis in Cardiovascular Toxicity

5-fluorouracil (5-FU) is a chemotherapy drug commonly used to treat head and neck cancer as well as breast cancer. However, its clinical use is often hampered by dose-dependent cardiotoxicity, which can lead to serious heart-related issues such as arrhythmia, angina, heart failure, and even acute myocardial infarction. Accumulating evidence has validated the pivotal role of ferroptosis in 5-FU-associated cardiac injury, primarily driven by disrupted iron homeostasis and excessive ROS accumulation [94]. Consistent in in vitro and in vivo cardiac models, 5-FU markedly induces ferroptotic damage, as characterized by elevated intracellular Fe^2+^ and malondialdehyde (MDA) levels, depleted GSH content, and impaired mitochondrial function [94,95]. Mechanistically, 5-FU exerts pro-ferroptotic effects by downregulating GPX4 and FTH1 while upregulating TFR1 [94]. This molecular alteration disrupts cellular iron balance, causes intracellular iron overload, and ultimately exacerbates lipid peroxidation and cardiac ferroptosis. Supporting the causal link between ferroptosis and 5-FU cardiotoxicity, the specific ferroptosis inhibitor ferrostatin-1 (Fer-1) effectively alleviates 5-FU-induced cardiac damage in vitro [94,95]. Subsequent studies have further supplemented the regulatory mechanisms underlying this pathological process. Fujii et al. identified an alternative mechanism of 5-FU-triggered cardiac ferroptosis: 5-FU dysregulates the expression of heme oxygenase-1 (HO-1) and ferrochelatase, leading to abnormal mitochondrial iron deposition and subsequent cardiac injury [95]. Moreover, Li et al. verified that resveratrol, a potent free radical scavenger, confers protective effects against 5-FU-induced cardiac dysfunction and oxidative stress. Both in vivo and in vitro experiments confirm that resveratrol ameliorates 5-FU-induced cardiotoxicity by suppressing GPX4-mediated ferroptosis [96].

### 4.3. HER2 Inhibitor-Induced Cardiac Ferroptosis

Human epidermal growth factor receptor 2 (HER2) inhibitors are a class of therapeutic drugs that specifically target HER2-positive breast cancer, such as trastuzumab, lapatinib, and neratinib. These medications can contribute to the development of cardiotoxicity by interfering with multiple pathways, including the neuregulin 1 pathway, oxidative stress, autophagy, and ferroptosis [97,98,99]. Clinical trials have revealed that the incidence of cardiac dysfunction among patients on trastuzumab monotherapy is approximately 3–7% [100]. Research involving trastuzumab-treated rats has revealed increased levels of lipid peroxidation and ACSL4, along with decreased expressions of GPX4, indicating heightened myocardial ferroptosis [101]. Mechanistically, trastuzumab suppresses the HER2/PI3K/AKT/NRF2 signaling cascade, which downregulates GPX4 and causes the accumulation of lipid peroxidation products, including 4-hydroxynonenal (4-HNE) and MDA. Mitochondrial 4-HNE further binds and promotes the oligomerization of voltage-dependent anion channel 1 (VDAC1), thereby inducing mitochondrial dysfunction. Moreover, trastuzumab perturbs mitochondrial GSH/GSSG balance and iron homeostasis, further destabilizing mitochondrial GPX4 [102]. Further pharmacological evidence confirms the pivotal role of ferroptosis in this pathological process. Ferroptosis inhibitors (e.g., Fer-1) and the iron chelator deferoxamine effectively ameliorate trastuzumab-induced cardiomyopathy by restoring PI3K/AKT/NRF2 signaling and inhibiting mitochondrial lipid peroxidation [102]. Complementarily, recent mechanistic studies have uncovered a transcription factor specificity protein 1 (SP1)-mediated regulatory pathway in trastuzumab-induced ferroptosis. Trastuzumab upregulates ACSL4 by activating the transcription factor SP1, which directly binds to the ACSL4 promoter, while simultaneously repressing the ferroptosis suppressor SLC7A11. Notably, SP1 silencing alleviates trastuzumab-triggered cardiac ferroptosis and lipid peroxidation, whereas Fer-1 co-treatment reverses these protective effects, validating the SP1/ACSL4 axis as a critical regulatory mechanism underlying trastuzumab-related cardiotoxicity [103]. Collectively, these findings solidify ferroptosis as a core mechanism responsible for trastuzumab-associated cardiotoxicity.

### 4.4. Tyrosine Kinase Inhibitor-Induced Ferroptosis in Cardiovascular Toxicity

Tyrosine kinase inhibitors are essential in treating various tumors, with sorafenib being the first FDA-approved inhibitor targeting the vascular endothelial growth factor (VEGF) pathway. Ferroptosis plays a critical role in enhancing the antitumor efficacy of sorafenib. Sorafenib suppresses the transcription of SLC7A11 by downregulating hypoxia-inducible factor 1-alpha (HIF-1α) expression or inhibiting NRF2 deacetylation via the protocadherin 20/sirtuin 1 (PCDH20–SIRT1) signaling pathway, which impairs the antioxidant defense of tumor cells and ultimately induces tumor cell ferroptosis [104,105]. Furthermore, research by Liu et al. have demonstrated that circular RNA cIARS is upregulated in sorafenib-treated HCC cells. The interaction between cIARS and the negative autophagy regulator AlkB Homolog 5, RNA demethylase (ALKBH5) promotes the dissociation and release of Beclin 1 from the B-cell lymphoma 2 (BCL2)/Beclin 1 complex, thereby inducing tumor cell ferroptosis through enhanced ferritinophagy [106]. However, it is important to note that due to the non-specific effects of sorafenib on both tumor cells and cardiomyocytes, its ferroptosis-dependent antitumor effects may concurrently promote cardiomyocyte ferroptosis [5]. With the extensive use of sorafenib in HCC and renal cell carcinoma, its cardiovascular-adverse effects have prompted increasing concern. Hypertension is the most common adverse event in patients under sorafenib treatment, resulting from endothelial function disruption and vascular remodeling caused by the inhibition of vascular endothelial growth factor receptor (VEGFR), platelet-derived growth factor (PDGF), and fibroblast growth factor receptor (FGFR) [107]. In addition, sorafenib treatment is associated with a range of severe cardiovascular complications, such as embolism and cardiac dysfunction [108,109]. A recent study has demonstrated that Krüppel-like transcription factor 11 activation can promote ferroptosis by suppressing FSP1 transcription during sorafenib-induced cardiotoxicity [110]. Additionally, Zhang et al. have reported that the level of activating transcription factor 3 (ATF3) is significantly increased in sorafenib-treated human cardiomyocytes. The downregulation of SLC7A11 mediated by ATF3, is identified as a crucial mechanism leading to sorafenib-induced cardiotoxicity [111].

### 4.5. ICI-Induced Cardiac Ferroptosis

Immune checkpoint inhibitors (ICIs) are designed to block the interactions between immune checkpoints, such as programmed cell death protein 1 (PD-1) and cytotoxic T-lymphocyte-associated protein 4 (CTLA-4), and their ligands, which in turn activates the immune system to target and eliminate tumor cells. Common ICIs include pembrolizumab (an anti-PD-1 therapy), ipilimumab (an anti-CTLA-4 therapy), and combination therapies (e.g., anti-PD-1 and anti-CTLA-4 dual immunotherapy). A multicenter study showed that the incidence of CTRCD among cancer patients receiving ICI treatment was 6.5% [112]. Among the cardiac immune-related adverse events induced by ICIs, myocarditis stands out as the most severe, marked by a notable infiltration of CD8^+^ cytotoxic T cells into the myocardium [113]. These activated CD8^+^ T cells can not only induce ferroptosis in tumor cells but also trigger ferroptosis in surrounding non-tumor cells via the action of IFN-γ. Despite this understanding, there is currently a lack of direct evidence linking ferroptosis to myocarditis induced by ICIs.

### 4.6. Radiation Therapy-Induced Cardiac Ferroptosis

Radiation therapy is utilized across a spectrum of malignancies, including breast, lung, and neck tumors, either as a standalone therapy or in conjunction with surgical and chemotherapeutic interventions. Due to the exposure of the heart within the radiation therapy field, cardiotoxicity remains a significant concern after radiotherapy, especially following chest radiotherapy. Acute cardiovascular complications may present as pericarditis and effusions, while common chronic complications include coronary artery disease and heart failure [114]. As early as the 1960s, investigators found that the iron content of organs increases after exposure to ionizing radiation due to tissue hemorrhage and heme degradation in local tissues [115]. This accumulated iron may serve as an initiator of ferroptosis in both normal and neoplastic tissues when subjected to gamma radiation. A recent study has highlighted that radiation-induced DDHD domain-containing 2 (DDHD2) downregulation promotes endothelial ferroptosis through the NRF2/GPX4 pathway, thereby accelerating atherosclerosis [116].

### 4.7. ER Stress–Ferroptosis Crosstalk in Cardiac Injury

Endoplasmic reticulum (ER) stress is a key upstream mediator of ferroptotic cardiovascular toxicity induced by anticancer therapies. A spectrum of anticancer agents, such as anthracyclines and tyrosine kinase inhibitors, disrupt cardiomyocyte ER homeostasis and initiate ferroptotic signaling through multiple intertwined molecular mechanisms [117,118,119]. Recent mechanistic study has uncovered the protein kinase RNA-like endoplasmic reticulum kinase (PERK)-mediated integrated stress response serves as a protective axis against ferroptosis by enhancing SLC7A11/GSH/GPX4 signaling in cardiomyocytes, representing an early adaptive reaction to ER perturbation [119]. Apart from the PERK pathway, the inositol-requiring enzyme 1/c-Jun N-terminal kinase (IRE1/JNK) axis exacerbates cardiomyocyte damage by promoting Drp1-mediated mitochondrial fission and ferritinophagy, ultimately facilitating ferroptosis progression [120]. Beyond these signaling cascades, ER stress disrupts mitochondria-associated ER membranes, perturbing mitochondrial homeostasis and elevating ROS levels amplify ferroptosis [121]. Moreover, ER stress-triggered mitochondrial DNA leakage activates the inflammatory cyclic GMP-AMP synthase-stimulator of interferon genes (cGAS-STING) pathway, which acts as an additional non-canonical regulatory mechanism that connects ER dysfunction to cardiomyocyte ferroptosis [118]. Collectively, ER stress acts as a vital mechanistic bridge linking anticancer therapy exposure to ferroptotic cardiac injury via the coordinated regulation of redox imbalance, mitochondrial dysfunction, and lipid peroxidation. These findings establish ER stress as a central regulatory node governing ferroptosis-mediated cardiotoxicity during cancer treatment.

### 4.8. Metabolic Regulation-Mediated Cardiac Ferroptosis

Given the inherent metabolic nature of ferroptosis, cardiomyocyte metabolism critically determines ferroptosis susceptibility. Cardiomyocytes are highly oxidative, relying on mitochondrial oxidative phosphorylation (OXPHOS) and fatty acid oxidation (FAO), which creates a ferroptosis-prone environment as mitochondria are both ROS sources and lipid peroxidation sites [122]. Fatty acid metabolism regulates ferroptosis sensitivity: ACSL4, LPCAT3, and CD36 promote ferroptosis by facilitating PUFA incorporation, whereas monounsaturated fatty acids confer resistance [122]. PPARα activation enhances FAO and transcriptionally upregulates GPX4, suppressing cardiac ferroptosis [123,124]. Beyond transcriptional regulation by PPARα, pathological FAO-to-glycolysis switching modulates ferroptosis sensitivity, and hypoxia/reoxygenation-induced glycolysis promotes ferroptosis via GPX4 lactylation at K218/K228 sites [125]. In parallel with glycolytic activation, OXPHOS disruption promotes ferroptosis; Methyltransferase-like 3 (METTL3)-mediated methionine sulfoxide reductase-B3 (MSRB3) m6A modification inhibits OXPHOS and accelerates ferroptosis, whereas METTL3 inhibition restores MSRB3 and alleviates dysfunction [126]. Collectively, these observations establish metabolism as a critical ferroptosis determinant, mechanistically linking metabolic perturbations to cardiomyocyte loss in cardiovascular diseases and anticancer therapy-related cardiotoxicity.

The therapeutic relevance of this metabolic framework is exemplified by anticancer therapy-induced cardiotoxicity. DOX disrupts myocardial iron homeostasis and induces lipid metabolic reprogramming, promoting peroxidation-prone substrate accumulation and ferroptotic cell death [127]. Trastuzumab suppresses HER2/PI3K/AKT/NRF2 signaling, downregulates GPX4, and promotes mitochondrial 4-HNE-mediated voltage-dependent anion channel 1 (VDAC1) oligomerization, driving ferroptosis-dependent mitochondrial dysfunction [102]. Lapatinib upregulates activating transcription factor 4 (ATF4) expression, suppresses GPX4, and triggers lipid peroxidation and ferroptosis in cardiomyocytes [128]. Sorafenib induces cardiomyocyte ferroptosis through the NRF2/HO-1/ferritin-mediated iron homeostasis disruption, whereas sunitinib acts through NRF2-dependent oxidative stress coupled with metabolic reprogramming toward glycolysis [129,130,131].

## 5. Emerging Targeted Strategies and Translational Progress

The emergence of cardio-oncology has driven the integration of multidisciplinary perspectives to strengthen cardiovascular risk management during cancer therapy. Ferroptosis, as a key mechanism underlying cardiovascular toxicity induced by various anticancer treatments, has emerged as a potential therapeutic target. This section systematically reviews the ferroptosis-modulating effects of clinical cardioprotective drugs, the preclinical progress of novel ferroptosis-specific inhibitors, and the emerging landscape of clinical biomarkers and ongoing trials, with the aim of bridging fundamental mechanistic insights with clinical translational applications. In addition to individual cardioprotective applications of ferroptosis-targeted approaches, drug interactions arising from combination regimens profoundly shape therapeutic outcomes and cardiovascular risks. We thus elaborate on these interactions and emphasize that dual primary endpoints are indispensable for developing ideal ferroptosis-centered cardio-oncological regimens.

### 5.1. Ferroptosis-Modulating Effects of Clinical Cardioprotective Drugs

DXZ, the sole FDA-approved drug for the prevention of anthracycline-induced cardiotoxicity, inhibits cardiomyocyte ferroptosis through iron chelation, thereby mitigating mitochondrial iron overload and lipid peroxidation [91,92]. Beyond DXZ, angiotensin-converting enzyme inhibitors (ACEI)/angiotensin receptor blockers (ARB) and β-blockers, first-line treatments for CTRCD, also modulate ferroptosis. Specifically, activation of angiotensin II type 1 and type 2 receptor (AT1/AT2) signaling provokes mitochondrial dysfunction and facilitates cardiomyocyte ferroptosis through the GSH–GPX4 and FSP1–CoQ10 axes; such detrimental effects can be abolished by angiotensin II type 1 and type 2 receptor (AT1/AT2) blockade [132]. Likewise, LCZ696 (sacubitril/valsartan) represses ferroptosis by activating the AKT/ sirtuin 3 (SIRT3)/superoxide dismutase 2 (SOD2) pathway and alleviates DOX-induced cardiotoxicity [133]. Among β-blockers, carvedilol elevates GPX4 and FTH1 expression to restrain lipid peroxidation, conferring protection against ferroptosis in hypoxia/reoxygenation-challenged cardiomyocytes [134,135]. Furthermore, SGLT2 inhibitors suppress cardiomyocyte ferroptosis in cardiac hypertrophy via upregulating GPX4 [136]. Similarly, vericiguat hinders cardiac ferroptosis and ameliorates DOX-induced cardiotoxicity by activating the SLC7A11/GPX4 axis [137]. Notably, statins exert context-dependent actions. By inhibiting HMG-CoA reductase and downregulating GPX4, statins initiate ferroptosis in tumor cells, which underpins their potential anti-tumor activity. On the contrary, statins confer cardioprotection by suppressing ferroptosis in preclinical models of myocardial ischemia–reperfusion injury and heart failure [138,139].

### 5.2. Novel Ferroptosis-Specific Inhibitors

Fer-1 is the most well-characterized specific inhibitor of ferroptosis. In mouse models of DIC, Fer-1 alleviates myocardial injury and preserves cardiac function. Furthermore, Fer-1 mitigates acute and chronic heart failure in myocardial ischemia–reperfusion injury models [140]. In addition, Fer-1 blocks angiotensin II-triggered myocardial remodeling and cardiac dysfunction in hypertensive mice via the interleukin-6 (IL-6)/STAT3/GPX4 pathway, and rescues ferroptosis-driven cardiomyopathy in cardiomyocyte-specific FTH1 knockout mice [24,141]. Beyond Fer-1, liproxstatin-1 has been shown to prevent diastolic dysfunction in diabetic cardiomyopathy [142]. Apart from these classic ferroptosis inhibitors, multiple novel small-molecule ferroptosis suppressors have recently been discovered. Herbacetin directly interacts with tyrosine 342 of ACSL4 to restrain lipid peroxidation and ameliorate DIC [143]. Dexmedetomidine exerts anti-ferroptotic effects through the AKT/glycogen synthase kinase (GSK)3β/NRF2 cascade, thereby alleviating DOX-induced cardiotoxicity [144]. Likewise, isoliquiritigenin attenuates myocardial ischaemia–reperfusion injury by modulating the NRF2/HO-1/SLC7A11/GPX4 axis [145].

### 5.3. Clinical Biomakers and Ongoing Trials

Preclinical and emerging exploratory clinical evidence supports the translational potential of ferroptosis-related biomarkers for early detection and monitoring of CTRCD. In models of DIC, the cornerstone of CTRCD research, ferroptosis markers such as MDA, 4-HNE, Fe^2+^, and GPX4 have been consistently validated as reliable indicators of myocardial injury [84,90,146]. Bioinformatics analyses integrating machine learning have identified Kelch domain-containing protein 3 (KLHDC3) and N-myc downstream-regulated gene 1 (NDRG1) as novel candidate biomarkers, with high diagnostic value validated in independent datasets [147]. At the clinical translational level, an exploratory trial (ChiCTR2200056796) demonstrated that GPX4 activation via selenomethionine-enriched mushroom supplementation reduced serum cardiac enzyme levels in breast cancer patients receiving DOX-based regimens, supporting the cardioprotective potential of targeting the ferroptosis pathway [84]. Despite these advances, large-scale prospective studies dedicated to ferroptosis in cancer therapy-related cardiotoxicity remain lacking. Overall, clinical translation of ferroptosis biomarkers in cardio-oncology remains nascent, hindered by the absence of standardized assay systems and safe targeted agents.

### 5.4. Ferroptosis Modulator-Related Drug Interactions in Cardio-Oncology

Ferroptosis modulator-related drug–drug interactions (DDIs) represent an emerging but poorly characterized determinant of both anticancer efficacy and cardiovascular safety. When combined with anticancer therapies, ferroptosis-targeting agents may differentially influence tumor response and treatment-related cardiotoxicity; however, integrated evaluation of these dual outcomes remains limited. A limited number of studies in tumor-bearing models have demonstrated the feasibility of efficacy-preserved cardioprotection. CDDO-Me alleviated DOX/lapatinib-induced cardiac ferroptosis without compromising antitumor efficacy [148]. Similarly, BRD4770 reduced DOX-induced cardiac ferroptosis and dysfunction while preserving DOX-mediated antitumor activity [149]. These findings suggest that selective ferroptosis modulation may provide cardiovascular protection without attenuating anticancer efficacy. However, most ferroptosis-related interventions have not been systematically evaluated using dual endpoints. Although the specific ferroptosis inhibitors Fer-1 and liproxstatin-1 effectively inhibit canonical ferroptosis, their effects on chemotherapy-associated cardiac injury appear to be context-dependent, and their impact on anticancer efficacy remains insufficiently characterized [150,151]. Future studies should integrate antitumor efficacy and cardiovascular toxicity as co-primary endpoints to identify ferroptosis-based strategies that preserve therapeutic antitumor efficacy while minimizing cardiovascular risk.

## 6. Conclusions

The emergence of cardio-oncology has driven the integration of multidisciplinary perspectives to enhance cardiovascular prevention, monitoring, and management during cancer treatment [82]. Although advances in anticancer therapies have markedly improved cancer survival, CTRCD remains a major challenge affecting long-term outcomes among cancer survivors [152]. Current preventive and therapeutic strategies have provided important clinical benefits; however, the complex molecular mechanisms underlying CTRCD remain incompletely understood. The shared risk factors and overlapping mechanistic pathways linking cardiovascular diseases and cancer are key contributors to cardiovascular injury stemming from anticancer interventions [153]. Among these mechanisms, ferroptosis has emerged as a critical regulatory process linking cancer therapy with cardiovascular toxicity.

Cancer therapy-related cardiovascular toxicity arises from a multitude of interconnected mechanisms, which include programmed cell death, inflammation, immune system activation, microvascular dysfunction with ischemia, and metabolic disturbances. Among these mechanisms, ferroptosis emerges as a critical factor, distinguished by iron dysregulation, compromised antioxidant defenses, and the abnormal accumulation of lipid peroxides, with cardiomyocytes demonstrating particular susceptibility to this process. The susceptibility of cardiomyocytes to ferroptosis under stress conditions can be attributed to their elevated mitochondrial density, cardiolipin-rich membranes containing PUFAs, and an iron-rich microenvironment. Accumulating evidence has established the involvement of ferroptosis in various cardiovascular pathologies, including myocardial ischemia–reperfusion injury, atherosclerosis, heart failure, and cancer therapy-related cardiovascular complications. This review elucidates the dual role of ferroptosis in tumorigenesis and CTRCD. While targeting ferroptosis represents a promising anticancer strategy, it is critical to recognize that biological consequences of ferroptosis within the TME and normal tissues require careful consideration. For instance, ferroptotic tumor cells can secrete KRASG12D to polarize TAMs toward a pro-tumor M2 phenotype. These findings highlight the complexity of ferroptosis-based therapeutic strategies and indicate that the clinical application of ferroptosis requires precise regulation to maximize antitumor efficacy while avoiding unintended biological consequences.

A major challenge in ferroptosis-targeted therapy is achieving sufficient tumor selectivity while preserving normal tissue integrity. The identification of ferroptosis-related biomarkers, tissue-specific ferroptosis regulatory mechanisms, and targeted delivery systems, may provide potential solutions [154,155]. For instance, a manganese-based nanoenzyme has been developed to synergize ferroptosis induction and immunotherapy, thereby enhancing theranostic efficacy in breast cancer [156]. DXZ, the only FDA-approved drug for preventing AIC, acts through ubiquitin/proteasome-mediated degradation of topoisomerase IIβ while sparing topoisomerase IIα, thereby reducing DNA double-strand breaks [157]. Meanwhile, emerging topoisomerase IIβ-selective strategies may provide additional cardioprotective opportunities while minimizing interference with anticancer efficacy [158]. Together, these advances emphasize that future ferroptosis-based therapies should focus on context-dependent regulation rather than global activation or inhibition.

Current research often focuses separately on ferroptosis in either oncology or cardiology. We advocate for integrated approaches that simultaneously address both aspects, facilitating the development of anticancer therapies with minimal cardiovascular toxicity. Future studies integrating tumor biology, cardiovascular metabolism, and ferroptosis regulation will be essential for establishing personalized cardio-oncology strategies and achieving a balance between effective cancer treatment and cardiovascular protection.

## Figures and Tables

**Table 1:** Summary of ferroptosis-dependent cardiotoxicity across anticancer modalities.

Drug/Treatment	Mechanism	Effect	Cardiovascular Diseases
Doxorubicin	GPX4 destabilization via FUNDC2/SLC25A11	Knockout of FUNDC2 ameliorates DOX-induced cardiomyopathy	Dose-dependent cardiomyopathy heart failure/arrhythmia
	Inhibit KEAP1/NRF2 /antioxidant genes	Induce cardiomyopathy	
	Extract Fe^3+^ from ferritin to form DOX-Fe^3+^	Induce cardiotoxicity	
	Inhibits mitochondrial iron export via ABCB8	ABCB8 exports DOX out of mitochondria and decreases DOX-induced cardiotoxicity	
	Increase mitochondrial iron influx via p53/Park7 /MFRN	Induce cardiomyocyte ferroptosis	
5-Fluorouracil	Iron homeostasis dysregulation ROS accumulation	Increase cardiac ferroptosis	Myocardial ischemia and angina
	GPX4 downregulation FTH1 downregulation TFR1 upregulation	Promote intracellular iron overload and lipid peroxidation	
	HO-1 alteration Ferrochelatase expression	Induce mitochondrial iron deposition and cardiac injury	
Trastuzumab	Inhibit HER2/PI3K/AKT /NRF2/GPX4	Promote lipid peroxidation and cardiac ferroptosis	Left ventricular dysfunction and heart failure
	Increase ACSL4 expression Suppress SLC7A11 via SP1 activation	Induce ferroptosis and myocardial injury	
	Promote 4-HNE-mediated VDAC1 oligomerization Mitochondrial dysfunction	Impair mitochondrial function and increase oxidative stress	
	Disrupt mitochondrial GSH/GSSG balance Disrupt iron homeostasis	Enhance mitochondrial ferroptosis susceptibility	
Sorafenib	Inhibit KLF11/FSP1 pathway	Induce cardiotoxicity	Hypertension/embolism/ cardiac dysfunction
	Inhibit ATF3/SLC7A11 pathway	Induce cardiotoxicity	
	Inhibit ATF4/SLC7A11 pathway	Induce cardiotoxicity	
Arsenic trioxide	Induce ROS and the loss of mitochondrial membrane potential	Induce ferroptosis in cardiomyoblast cells	QT prolongation and Torsades de Pointes
ICI	CD8^+^ cytotoxic T-cell infiltration	Induce tumor cell ferroptosis may also cause cardiac ferroptosis	Immune-mediated myocarditis
Radiation therapy	Inhibit DDHD2/NRF2 /GPX4 pathway	Induce endothelial ferroptosis and atherosclerosis	Pericardial disease/coronary artery diseases
	Promote iron accumulation	Induce cardiomyocyte ferroptosis	

Footer: GPX4: Glutathione peroxidase 4; FUNDC2: FUN14 Domain-Containing 2; SLC25A11: Solute carrier family 25 member 11; DOX: Doxorubicin; KEAP1: Kelch-like ECH-associated protein 1; NRF2: Nuclear factor erythroid 2-related factor 2; Fe^3+^: Ferric iron; ABCB8: ATP-binding cassette subfamily B member 8; p53-tumor protein p53; Park7: Parkinson’s disease protein 7; MFRN: Mitoferrin; ROS: Reactive oxygen species; FTH1: Ferritin heavy chain 1; TFR1: Transferrin receptor; HO-1: Heme oxygenase-1; HER2: Human epidermal growth factor receptor 2; PI3K: Phosphoinositide 3-kinase; AKT: AKT serine/threonine kinase; ACSL4: Acyl-coenzyme A synthetase long-chain family member 4; SP1: Specificity protein 1; FSP1: Ferroptosis suppressor protein 1; HNE: Hydroxynonenal; VDAC1: Voltage-dependent anion channel 1; GSH: Glutathione; GSSG: Oxidized glutathione; KLF11: Krüppel-like transcription factor 11; ATF3: Activating transcription factor 3; SLC7A11: Solute carrier family 7 member 11; ATF4: Activating transcription factor 4; QT: QT interval: ICI: Immune checkpoint inhibitor; DDHD2: DDHD domain-containing 2.

## Data Availability

No new data were created or analyzed in this study. Data sharing is not applicable to this article.

## Abbreviations

The following abbreviations are used in this manuscript:

CTRCD	Cancer therapy-related cardiac dysfunction
LIP	Labile iron pool
ROS	Reactive oxygen species
PUFA	Polyunsaturated fatty acid
GPX4	Glutathione peroxidase 4
GSH	Glutathione
FSP1	Ferroptosis suppressor protein 1
CoQ10	Ubiquinone
GCH1	Guanosine-5′-triphosphate cyclohydrolase-1
BH4	Tetrahydrobiopterin
DHODH	Dihydroorotate dehydrogenase
CoQH_2_	Ubiquinol
CoA	Coenzyme A
Fe^3+^	Ferric iron
Fe^2+^	Ferrous iron
TFR1	Transferrin receptor
FTH1	Ferritin heavy chain 1
SLC7A11	Solute carrier family 7 member 11
Fpn	Ferroportin 1
AA	Arachidonic acid
ACSL4	Acyl-coenzyme A synthetase long-chain family member 4
IFN-γ	Interferon-γ
ALOX	Arachidonate lipoxygenase
PE	Phosphatidylethanolamine
CoQ	coenzyme Q
MVA	mevalonate
TME	Tumor microenvironment
p53	Tumor Protein p53
NRF2	Nuclear factor erythroid 2-related factor 2
TAMs	Tumor-associated macrophages
HCC	Hepatocellular carcinoma
KEAP1	Kelch-like ECH-associated protein 1
DOX	Doxorubicin
DIC	Doxorubicin-induced cardiomyopathy
DXZ	Dexrazoxane
FDA	Food and Drug Administration
5-FU	5-Fluorouracil
Fer-1	Ferrostatin-1
MDA	Malondialdehyde
HER2	Human epidermal growth factor receptor 2
4-HNE	4-hydroxynonenal
ICIs	Immune checkpoint inhibitors
PD-1	Programmed cell death protein 1
CTLA-4	Cytotoxic T-lymphocyte-associated protein 4
